# Evaluation of an identification method for the SARS-CoV-2 Delta variant based on the amplification-refractory mutation system

**DOI:** 10.3389/fcimb.2023.1180297

**Published:** 2023-07-05

**Authors:** Qin Zhang, Runjie Qiao, Jiaojiao Niu, Xia Xiong, Nan Wang, Ruixian Zhang, Sha Luo, Yuwan Guo, Zhonghua Liu, Li Peng, Shaoduo Zhang, Guolei Tan, Keyu Song, Mei Sun, Lulu Xu, Rong Zhang, Xuping Wu

**Affiliations:** ^1^The Second Hospital of Nanjing, Nanjing University of Chinese Medicine, Nanjing, Jiangsu, China; ^2^Research and Development Department, Jiangsu Bioperfectus Technologies Company Limited, Taizhou, Jiangsu, China

**Keywords:** SARS-CoV-2, Delta variant, amplification-refractory mutation system, variant identification, molecular identification

## Abstract

The Delta variant of SARS-CoV-2 dominated the COVID-19 pandemic due to its high viral replication capacity and immune evasion, causing massive outbreaks of cases, hospitalizations, and deaths. Currently, variant identification is performed mainly by sequencing. However, the high requirements for equipment and operators as well as its high cost have limited its application in underdeveloped regions. To achieve an economical and rapid method of variant identification suitable for undeveloped areas, we applied an amplification-refractory mutation system (ARMS) based on PCR for the detection of novel coronavirus variants. The results showed that this method could be finished in 90 min and detect as few as 500 copies/mL and not react with SARS-Coronavirus, influenza *A H1N1(2009)*, and other cross-pathogens or be influenced by fresh human blood, α- interferon, and other interfering substances. In a set of double-blind trials, tests of 262 samples obtained from patients confirmed with Delta variant infection revealed that our method was able to accurately identify the Delta variant with high sensitivity and specificity. In conclusion, the ARMS-PCR method applied in Delta variant identification is rapid, sensitive, specific, economical, and suitable for undeveloped areas. In our future study, ARMS-PCR will be further applied in the identification of other variants, such as Omicron.

## 1 Introduction

The emergence of novel coronavirus variants has been continuously reported since late 2019, including Alpha, Beta, Delta, Gamma, *etc* (Cherian et al., 2021; Grabowski et al., 2021; Singh et al., 2021; Tegally et al., 2021; Vaidyanathan, 2021; da Silva et al., 2022). Alpha, with a key mutation-N501Y in the receptor binding domain (RBD), was first detected in the UK in late 2020 and became the dominant variant in early 2021 (Davies et al., 2021). The Delta variant was first found in India in late 2020 and displaced Alpha as the dominant variant in mid-2021, leading to a resurgence of COVID-19 cases in many countries (Centers for Disease Control and Prevention, 2021; Bolze et al., 2022). A study from the US showed that the Delta variant was more transmissible than previous variants in part because of the higher viral load it caused during acute infection (Earnest et al., 2022). Another earlier study also reported that the Delta variant had a higher replication rate and immune escaping ability (Mlcochova et al., 2021). The Delta variant contains RBD mutation L452R and the proximal furin cleavage site mutation P681R (Tao et al., 2021). In addition to L452R and P681R, Kappa (B.1.617.1), which shares a common ancestor with Delta (B.1.617.2), also has an additional RBD E484Q mutation (Tao et al., 2021). L452R and E484Q mutations increased the interaction between the spike protein and ACE2, possibly leading to increased viral infectivity and immune escape (Cherian et al., 2021; Deng et al., 2021; Augusto et al., 2022). P681R mutation increased the cutting rate between S1/S2, increasing virus replication and ultimately resulting in higher transmissibility (Hoffmann et al., 2020; Cherian et al., 2021; Liu et al., 2022). Moreover, the Delta variant posed the great risk to countries with limited access to vaccines, particularly in Africa, where the vaccination coverage in most countries was less than 5% of their population (Callaway, 2021). Reduced sensitivity of the Delta variant to antibody neutralization and the lower effectiveness of vaccines after the receipt of the first dose against the Delta variant were reported (Liu et al., 2021; Lopez Bernal et al., 2021; Planas et al., 2021).

Due to the increased prevalence and potential dangers posed by the Delta variant, there is a critical need for a more effective method of detection. Real-time reverse transcription PCR, the most common method used to detect SARS-CoV-2, and other methods which have been developed, such as RT-LAMP and CRISPR, could determine only whether samples contain SARS-CoV-2 but do not identify the type of variants (Broughton et al., 2020; Corman et al., 2020; Dao Thi et al., 2020). Currently, the identification of virus variants relies mainly on whole-genome sequencing, in which the nucleotide sequences of unknown samples are obtained and compared with known pathogen sequences (Charre et al., 2020; Lu et al., 2020). However, whole-genome sequencing requires the availability of highly professional instruments, high testing costs, long testing time, and complex results analysis. These difficulties and deficiencies have limited its application in undeveloped areas, which cannot currently meet the requirements of rapid virus variant identification. In this regard, Aoki et al. developed a genotyping platform for SARS-CoV-2 variant identification using high-resolution melting analysis, but it has not been practically tested on clinical samples of COVID-19 patients (Aoki et al., 2021). Doubtless, the development of multiple platforms and methods that can detect the type of SARS-CoV-2 variants is highly necessary.

Amplification-refractory mutation system (ARMS) is a single, rapid, and reliable method for detecting any mutation involving single-base changes or small deletions (Newton et al., 1989; Little, 2001). It is commonly used to perform tests for point mutations in a variety of genetic diseases and cancers, such as phenylketonuria, thalassemia, and *BRAF* V600E mutation (Huang et al., 2013; Shaykholeslam Esfahani et al., 2018; Gholami et al., 2021). In this study, ARMS was established for detecting the Delta variant, which overcomes the shortcomings of sequencing. The assay was successfully tested on 262 clinical samples from patients infected with the Delta variant.

## 2 Materials and methods

### 2.1 Plasmid construction and synthesis of primers and probes

The combination mutations of L452R and P681R (nucleotide positions T22917G and C23604G) could differentiate B.1.617 lineage from other variants. The E484Q (nucleotide position G23012C) mutation could differentiate other variants from Delta among B.1.617 lineage. We designed three pairs of mutant primers for these three mutant sites. Primers and probes used in this assay were synthesized from Shuoying Biotechnology (Shanghai, China). Negative control consisted of a plasmid containing fragments of internal standard (RNaseP). Both positive control and negative control were synthesized by GeneoDx Biotech Co., LTD (Shanghai, China). The virus-like particles (VLPs) used to determine diagnostic cut-off values were ordered by Shuoying Biotechnology (Shanghai, China). The sequences of the positive and negative control are displayed in **Table S1**. The sequences of the primers and probes of L452R, E484Q, P681R mutations, *ORF1ab* gene, and RNaseP used in this experiment are presented in **Table 1**.

**Table 1:** Details of probes and primers sequences used in ARMS-PCR assay.

Oligonucleotide function	Oligonucleotide name	Sequence^*^	Length &Tm^**^
Forward primer for L452R	L452R-F	5′ AGGTTGGTGGTAATTATAATTAAC**G**	25nt 56.9°C
Reverse primer for L452R	L452R-R	5′ GCTACCGGCCTGATAGATTT	20nt 56.4°C
Probe for L452R	L452R-P	5′ ^‡^FAM-TATCTCTCTCAAAAGGTT-MGB	18nt 66°C
Forward primer for E484Q	E484Q-F	5′ GCACACCTTGTAATGGTGAT**C**	21nt 55.1°C
Reverse primer for E484Q	E484Q-R	5′ CAGTTGCTGGTGCATGTAGAA	21nt 57.1°C
Probe for E484Q	E484Q-P	5′ VIC-TCAAAAGAAAGTACTACTAC-MGB	20nt 64°C
Forward primer for P681R	P681R-F	5′ AGTTATCAGACTCAGACTAATTCTC**G**	26nt 56°C
Reverse primer for P681R	P681R-R	5′ CACCAAGTGACATAGTGTAGGCA	23nt 57.9°C
Probe for P681R	P681R-P	5′ VIC-CGGCGGGCACGTAGTGTAGCTAG-BHQ	23nt 68°C
Forward primer for ORF1ab	ORF1ab-F	5′ CTTTGGCTTGATGACGTAGTTT	22nt 56.8°C
Reverse primer for ORF1ab	ORF1ab-R	5′ TGAGTAAATCTTCATAATTAGGGTT	25nt 54.5°C
Probe for ORF1ab	ORF1ab-P	5′ FAM-CTTCAGAGGTGCAGATCACATGTC-BHQ	24nt 61.2°C
Forward primer for RNaseP	RNaseP-F	5′ ACAGGGAAAATCAAGACCAAT	21nt 55.5°C
Reverse primer for RNaseP	RNaseP-R	5′ TCAAAACATTGCAGTGAGATGGA	23nt 60.7°C
Probe for RNaseP	RNaseP-P	5′ CY5-ATTTTAACTAGATTAACAATTATTGTCTCGG-BHQ	31nt 61.4°C

^*^The terminal 3′ nucleotide being targeted is highlighted in bold italic. ^**^Tm calculated using Primer Premier 5 software tool with qPCR parameter set. ^‡^ FAM = 6-Carboxyfluorescein. MGB, Minor Groove Binder; BHQ, Black Hole Quencher; VIC, 5-VIC phosphoramidite; CY5, Cyanine 5.

### 2.2 Amplification-refractory mutation system-PCR assay

**Figure 1:**
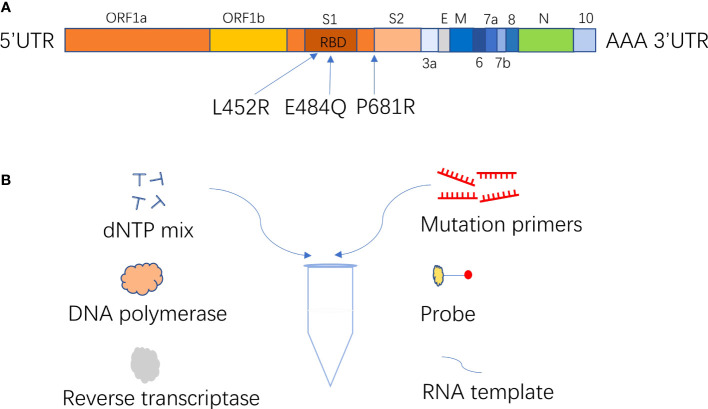
**(A)** Location of major mutation site of B.1.617; **(B)** ARMS-PCR diagrammatic diagram.

The ARMS-PCR assay, based on an amplification-refractory mutation system and real-time fluorescence PCR technology, was developed for the identification of Delta variant infection (**Figure 1**). In this experiment, *S-*gene was used as the target sequence to design mutant primers of L452R, E484Q, and P681R; ORF1ab gene was used as the target sequence to design internal control primer. The probes were labeled with different fluorescence to detect multiple targets in one reaction tube (**Table 1**). The mutant primers were efficient only for the mutation sequence amplification, while wild-type sequence greatly reduced the amplification efficiency, and even no amplification because of the mismatch between primers and bases. The fluorescence quantitative PCR instrument could automatically draw a real-time amplification curve based on the detected fluorescence signal thus achieving the mutation detection of L452R, E484Q, and P681R mutation sites of coronavirus *S*-gene according to the difference of Ct value of mutation and *ORF1ab* gene reaction (ΔCt). In this study, each sample was distributed in two reaction tubes, tube A and tube B. Reaction A mixture contained primers and probes of RNaseP and mutations L452R and P681R on the *S*-gene. Reaction B mixture contained primers and probes of the *ORF1ab* gene and mutation E484Q on the *S*-gene. ARMS-PCR was performed in a final mixture with a volume of 25 uL containing 5 uL of the sample, 7.5 uL of detection solution, 7.5 uL of nucleic acid amplification reaction solution, and 5 uL of enzyme mixture. The specific concentrations of the primers and probes are presented in **Table S2**. The high concentration of the primer and probe at the E484Q site was to obtain sufficient fluorescence values during the reaction. All amplification reactions were performed on an ABI 7500 fluorescence quantitative PCR instrument (Thermo Fisher Scientific, USA). The PCR reaction was carried out in three stages: at 50°C for 10 min, 97°C for 1 min, 45 cycles of 97°C for 5 sec, and 58°C for 30 sec (fluorescence collection).

### 2.3 Data interpretation

**Figure 2:**
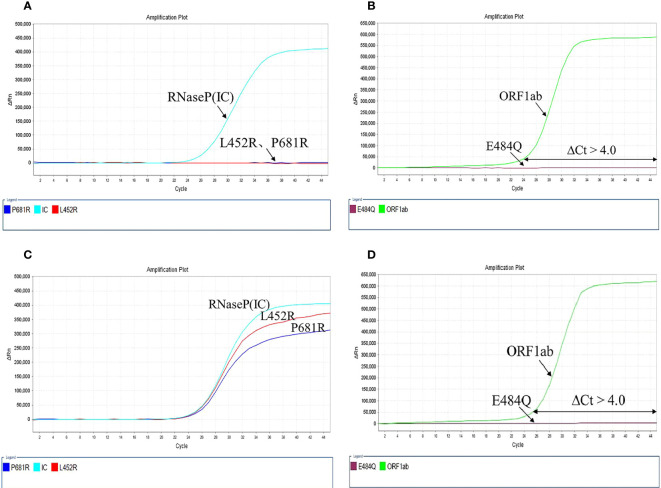
The results of the mutation test. **(A, B)** Non-B.1.617 mutant, L452R, P681R, and E484Q showed no mutation; **(C, D)** B1.617.2 mutant, L452R, P681R mutations with E484Q wild-type.

This method was designed mainly for variant identification rather than quantification. Therefore, calibration curves were not required. Calculate ΔCt of L452R, P681R, and E484Q mutation sites. ΔCt of the mutation X of a sample equals the Ct value of the mutation X (Mx) minus the Ct value of the ORF1ab: ΔCt(x) = Ct(Mx) – Ct(ORF1ab); ΔCt can be negative. If neither reaction produced Ct values in less than 40 cycles, it was interpreted as “no SARS-COV-2 detected.” The combination of mutations L452R and P681R were preliminarily classified into B.1.617 lineage. If the sample had additional mutation E484Q, it would be identified as B.1.617.1/3. Conversely, if the sample excluded the E484Q mutation, it would be identified as Delta variant (B.1.617.2). The test results of non-B.1.617 mutant samples and B.1.617.2 mutant samples can be seen in **Figure 2**.

### 2.4 Determination of the cut-off values and cut-off ΔCt values

The diagnostic cut-off values of the L452R, E484Q, and P681R mutations and the *ORF1ab* gene on the *S*-gene of SARS-CoV-2 in our assay were evaluated based on the Ct values of 200 simulated samples including 100 cases of VLPs-simulated samples and 100 cases of negative samples using Receiver Operating Characteristic (ROC) curve in SPSS19.0 software. ROC curve is an effective method to evaluate the performance of diagnostic tests, which is defined as a plot of test sensitivity as the *y* coordinate versus its 1-specificity or false positive rate as the *x* coordinate (Park et al., 2004). The determination of the diagnostic cut-off ΔCt values was based on the ΔCt values results of 100 VLPs-simulated samples and 100 cases of negative samples using ROC curves in SPSS19.0 software.

### 2.5 Sensitivity of the ARMS-PCR assay

Limit of detection (LOD) – The lowest amount of analyte in a sample that can be detected with (stated) probability, although perhaps not quantified as an exact value (revised from WHO-BS/95.1793). In this study, the lowest viral concentration level with a positive detection rate of 95% or greater was defined as the LOD. To determine the LOD of this method, we collected three types of samples, including nasopharyngeal swabs, throat swabs, and sputum samples. First, two nasopharyngeal swabs, two throat swabs, and two sputum samples were 10-fold serially diluted in five concentrations: at 5 × 10^5^ copies/mL, 5 × 10^4^ copies/mL, 5 × 10^3^ copies/mL, 5 × 10^2^ copies/mL, and 5 × 10^1^ copies/mL, and each dilution was tested by one lot of reagents for 20 repeats. Then, we determined the concentration in the nasopharyngeal swabs, throat swabs, and sputum samples detected with a positive rate higher than 95% as the LOD. Directly count the number of positive results corresponding to each dilution and calculated the positive detection rate (Number of positive results (n)/Number of total repeated tests of each concentration level (N)). Next, two positive nasopharyngeal swabs, two positive throat swabs, and two positive sputum samples from different patients were diluted to the LOD concentration, and each dilution was tested with three lots of reagents for 20 repeats. The LOD was verified by a positive rate of at least 19/20.

### 2.6 Specificity of the ARMS-PCR assay

To determine the specificity of the assay, we performed interference tests involving the cross-reactivity of pathogens which produce similar symptoms as SARS-CoV-2 and also involving potential endogenous and exogenous interfering substances. Samples in the cross-reactivity assay were prepared by adding cultured isolates or nucleic acid of the cross-pathogens (as shown in **Table S3**) into 37 low-concentration VLPs-simulated samples (3 × LOD) and 37 negative samples, respectively. Samples in interference testing were prepared by adding 4 potential endogenous and 6 exogenous interfering substances (as shown in **Table S4**) into 10 low-concentration VLPs-simulated samples (3 × LOD) and 10 negative samples, respectively. Each cross-pathogen or interfering substance was tested three times in low-concentration and negative samples. The experiments were performed on a QuantStudio™ 5 real-time PCR (Thermo Fisher Scientific, USA) instrument with one lot of reagents.

### 2.7 Collection of clinical sample and extraction of nucleic acid

The clinical samples collected in this study were all obtained from one epidemic (Nanjing Lukou Airport epidemic). Based on the epidemiological investigation and sequencing data, the local CDC (Nanjing CDC) announced that this outbreak was caused by the spread of the Delta variant. On admission, all the patients were identified to have Delta infection. All samples analyzed in this study were from patients infected with the Delta variant. A total number of 262 clinical samples (nasopharyngeal swabs, n=249; throat swabs, n=9; sputum, n=4) were collected from patients diagnosed with Delta variant infection at the Second Hospital of Nanjing, a designated hospital for COVID-19 treatment in Nanjing, China. This study was approved by the Medical Ethics Committee of the Second Hospital of Nanjing (2021-LS-ky037). The nucleic acid extraction was performed on an automatic nucleic acid extraction instrument (Bioperfectus diagnostics, Jiangsu, China) using a nucleic acid isolation kit (magnetic beads) (Bioperfectus diagnostics, Jiangsu, China) following the manufacturer’s instructions. After extraction, RNA was eluted in 60 µL of RNase-free water and stored at -80°C for later use. The interference samples used in this study were provided by Taizhou Center for Disease Control and Prevention (Taizhou, Jiangsu, China).

### 2.8 RT-PCR assay for SARS-CoV-2 detection

The assay for detecting SARS-CoV-2 was performed with a commercial RT-PCR fluorescence diagnostic kit (Sansure Biotech, China), which was approved by the Chinese National Medical Products Administration (CNMPA), Food and Drug Administration (FDA), and Conformité Européenne (CE) Certification. This commercial kit has been widely used to detect SARS-CoV-2 in hospitals, CDCs, and third-party detection institutions in China. A Slan-96s real-time PCR machine (Hongshi, China) was used to detect and a Ct value below 40 was considered positive. SARS-CoV-2 RNA was mixed with amplification mixture and put into Slan-96s for reaction at 50°C for 30 min, 95°C for 1 min, and 45 cycles of 95°C for 15 sec, 60°C for 30 sec, and 25°C for 10 sec.

## 3 Results

### 3.1 The demographic characteristics of 262 patients diagnosed with Delta variant infection

The 262 patients (158 females and 104 males) were clinically classified as 51 mild, 185 moderate, 19 severe, and 7 critical, as shown in **Table 2**. There was no significant difference in gender distribution, however, the age distribution showed an obvious difference. The average age was 48.61 ± 18.72. Patients older than 50 were more likely to develop into moderate, severe, and critical cases, while the ages of critical cases were all above 50. Besides, patients with basic diseases were more likely to develop into severe and critical cases. Hypertension and diabetes were the main basic diseases.

**Table 2:** Demographic characteristics in 262 patients with Delta variant infection.

Parameters	mild (n=51)	moderate (n=185)	Severe (n=19)	Critical (n=7)	All (n=262)	*p*
Gender						.132
Female	25 (49.02%)	120 (64.86%)	10 (52.63%)	3 (42.86%)	158 (60.31%)	
Male	26 (50.98%)	65 (35.14%)	9 (47.37%)	4 (57.14%)	104 (39.69%)	
Age, year	31.98 ± 18.02	51.49 ± 16.74	55.74 ± 10.76	74.43 ± 7.37	48.61 ± 18.72	.000
Age Group						.000
<50	43 (84.31%)	84 (45.41%)	6 (31.58%)	0 (0.00%)	133 (50.76%)	
≥50	8 (15.69%)	101 (54.59%)	13 (68.42%)	7 (100.00%)	129 (49.24%)	
Basic Diseases						
Hypertension	3 (50.00%)	46 (62.16%)	4 (40.00%)	4 (66.67%)	57 (59.38%)	.002
Diabetes mellitus	1 (16.67%)	15 (20.27%)	6 (60.00%)	2 (33.33%)	24 (25.00%)	.002
Surgical history	1 (16.67%)	11 (14.86%)	3 (30.00%)	2 (33.33%)	17 (17.71%)	.054
Tumor history	1 (16.67%)	8 (10.81%)	2 (20.00%)	1 (16.67%)	12 (12.50%)	.366
Cardiovascular disease	0 (0.00%)	3 (4.05%)	0 (0.00%)	1 (16.67%)	4 (4.17%)	.174

p <0.05 indicates statistical significance. Age, hypertension, and diabetes mellitus indicate statistical significance.

### 3.2 Confirmation of cut-off values and cut-off ΔCt values

**Figure 3:**
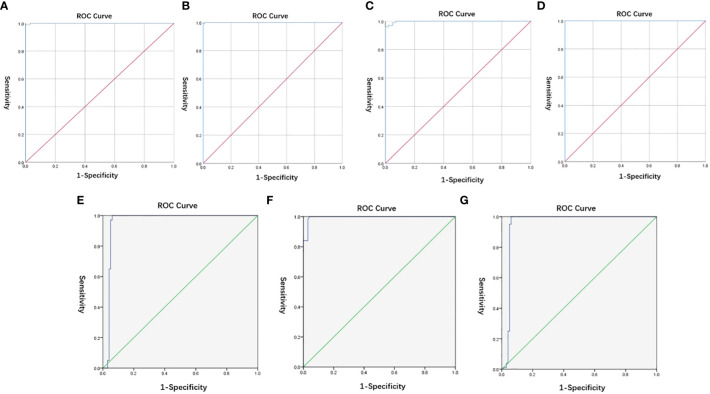
**(A–D)** ROC of cut-off values of L452R, E484Q, and P681R mutations on the *S*-gene and *ORF1ab* gene of SARS-CoV-2; **(E-G)** ROC of cut-off ΔCt values of L452R, E484Q, and P681R mutations on the *S*-gene of SARS-CoV-2.

The diagnostic cut-off values of L452R, E484Q, and P681R mutations on the *S*-gene and *ORF1ab* gene of SARS-CoV-2 were all equal to 40.0 to ensure high sensitivity based on ROC curves, as can be seen in **Figures 3A–D** and **Table S5** (displaying the Youden’s index). Similarly, the diagnostic cut-off ΔCt values of L452R, E484Q, and P681R mutations on the *S*-gene of SARS-CoV-2 were determined as 10.0, 4.0, and 10.0, respectively, (**Figures 3E–G**; **Table S6**).

### 3.3 Methodological sensitivity and specificity of ARMS-PCR assay

Through 20 replicates performed on the five series of concentrations diluted samples, we found that a concentration of 5 × 10^2^ copies/mL and above could be detected with a positive rate higher than 95% (**Table S7**). Thus, we determined 5 × 10^2^ copies/mL as the LOD of this assay. Then, we further verified the LOD by testing two nasopharyngeal swabs, two throat swabs, and two sputum samples at a concentration of 5 × 10^2^ copies/mL by three lots of reagents for 20 replicates. As visible in **Table 3**, three different types of samples were subjected to analysis with a positive detection rate higher than 95%. Therefore, the suggested here assay can detect as few as 5 × 10^2^ copies/mL; hence, 5 × 10^2^ copies/mL was determined and verified as the LOD in this study.

**Table 3:** Verification of LOD by nasopharyngeal swabs, throat swabs, and sputum.

Concentration	Sample ID	Positive results (n)/Negative results(n)			Total test (N)	Positive rate (%)
		Lot 1	Lot 2	Lot 3		
5 × 10^2^ (copies/mL)	Sample 1	19/1	20/0	20/0	60	98.3%
	Sample 2	20/0	20/0	20/0	60	100%
	Sample 3	20/0	19/1	19/1	60	96.7%
	Sample 4	20/0	20/0	19/1	60	98.3%
	Sample 5	20/0	20/0	20/0	60	100%
	Sample 6	20/0	20/0	20/0	60	100%

Sample 1 and 2 were nasopharyngeal swabs, sample 3 and 4 were throat swabs, sample 5 and 6 were sputum.

As can be seen in **Table S8**, there was no cross-reaction with the cross-pathogens in negative samples and VLPs-simulated samples (3 × LOD) were also undisturbed by cross-pathogens at the test concentration. The potential endogenous and exogenous interfering substances in negative samples and VLPs-simulated samples (3 × LOD) at the test concentration did not interfere with the detection (**Table 4**). Therefore, the proposed approach has very good specificity which is not influenced by cross-pathogens and interfering substances.

**Table 4:** Results of testing with potential endogenous and exogenous interfering substances.

Interfering substances	Concentration	Result (No. Positive/ No. Tested)		Result
		Negative samples (with interfering substances)	3× LOD (with interfering substances)	
Fresh human blood	5%	0/3	3/3	No reaction
Nasal secretions	5%	0/3	3/3	No reaction
Mucus	5%	0/3	3/3	No reaction
Mucoprotein	2g/dL	0/3	3/3	No reaction
α- interferon	100 units/mL	0/3	3/3	No reaction
Zanamivir	5mg/L	0/3	3/3	No reaction
Ribavirin	0.2g/L	0/3	3/3	No reaction
Mupirocin	0.2%	0/3	3/3	No reaction
Aspirin	50mg/L	0/3	3/3	No reaction
Tobramycin	10mg/L	0/3	3/3	No reaction

### 3.4 Comparison of ARMS-PCR and RT-PCR in clinical samples

After determining the sensitivity and specificity of ARMS-PCR, we further verified its performance in clinical samples. In a set of double-blind trials, we tested 262 clinical samples using both the RT-PCR kit (Sansure Biotech, China) and the ARMS-PCR assay to analyze and compare the differences between the two methods. As visible in **Table 5**, 245 positive samples and 17 negative samples were detected in the test results of the gold standard RT-PCR. The ARMS-PCR assay tested 235 positives and 27 negatives, of which 10 showed inconsistent results. Among the 17 samples with negative RT-PCR detection, ARMS-PCR detection also tested negative, showing good consistency. To analyze the difference in the test results between the two methods, we further evaluated and compared the performance of ARMS-PCR and RT-PCR in clinical positive samples (**Table 6**). To intuitively analyze the corresponding results of different Ct values, we divided these positive samples into three groups: a high group with Ct < 20, a middle group with 20≤Ct < 30, and a low group with Ct ≥ 30. Further analysis revealed that the Ct values of the samples with no results detected by ARMS-PCR were concentrated above the value of 35 (mainly distributed around 39), which meant that these samples had a low viral load (**Table S9**).

**Table 5:** The results of ARMS-PCR and RT-PCR in 262 clinical samples, of which 10 showed inconsistent results.

		Results of RT-PCR		Total
		+	-	
Results of ARMS-PCR	+	235	0	235
	–	10	17	27
Total		245	17	262

**Table 6:** The performance between ARMS-PCR and RT-PCR for detection of Delta variant in clinical positive samples.

Samples		ARMS-PCR	
		+	-
High (n=38)	+	38	0
	–	0	0
Middle (n=134)	+	134	0
	–	0	0
Low (n=73)	+	63	10^*^
	–	0	0
Total (n=245)	+	235	10
	–	0	0

10^*^: The Ct values of 10 samples with unidentified type were all greater than 35, indicating that the viral load of these samples was very low.

### 3.5 Results of the type identification of clinical samples by ARMS-PCR

Based on the data communicated at the press conference held by the local CDC, the transmission variant of this outbreak was identified as the Delta variant by sequencing. The samples used in our experiment were all obtained from COVID-19 patients with Delta variant infection that had been admitted to designated hospitals during the epidemic. As can be observed in **Table 7**, both L452R and P681R mutations were detected in all 235 clinical positive samples, but E484Q mutations were not detected in any of the 235 samples. Therefore, according to the explanation of this method, all 235 samples tested were identified as Delta variant (B.1.617.2), which was consistent with the epidemic findings reported.

**Table 7:** The results of type identification of ARMS-PCR in 235 clinical positive samples.

Results	Mutation site		
	L452R	E484Q	P681R
Mutation	235	0	235
Wild	0	235	0

## 4 Discussion

The Delta variant has higher transmissibility and pathogenicity than other variants, resulting in higher rates of hospitalization and mortality. The resurgence of the epidemic put a serious negative impact on the economy and social health welfare. The impact of the outbreak may be minimized by slowing the spread of variants in the bud. At this point, rapid tests play an important role in controlling variant spread. However, commercial rapid detection of SARS-CoV-2 antigens and antibodies has some shortcomings and cannot meet the need for accurate and rapid detection. Antigen tests are often less sensitive than nucleic acid tests due to the lack of amplification (Lai and Lam, 2021). Cross-reactivity with other pathogens causing false positives is a problem of antigen-antibody testing, but nucleic acid testing with specific primers and probes does not present this concern (Lustig et al., 2021). The lack of identification of variants is the critical problem of antigen and antibody tests. Therefore, it is necessary to develop multiple methods based on nucleic acid testing for variant identification.

In this experiment, specific mutant primers were designed for mutation sites L452R, P681R, and E484Q. The base mismatch between the primer and the template could effectively inhibit PCR reaction, thereby achieving the purpose of template differentiation. Because a single-base mutation could not cause a significant difference, an additional mismatch in some loci was introduced to ensure that chain extension could be effectively prevented. It is worth mentioning that we used internal standards (RNaseP) to monitor the collection, transportation, and extraction process of test samples during the experiment to avoid false negative results.

Several ARMS-PCR assays have been developed for disease diagnosis. For example, Esfahani et al. introduced a novel compound-primed multiplex ARMS-PCR (CPMAP) for simultaneous detection of common PAH gene mutations (Shaykholeslam Esfahani et al., 2018). Additionally, Huang et al. developed a method using an ARMS-PCR to detect the *BRAF* V600E mutation in formalin-fixed, paraffin-embedded (FFPE) tissue (Huang et al., 2013). The results of these studies indicate that ARMS-PCR has great potential in the diagnosis of diseases. In the present study, the ARMS-PCR assay could detect as few as 500 copies/mL of SARS-CoV-2 RNA and showed no cross-reaction with other cross pathogens, such as SARS-coronavirus, MERS-coronavirus, *Influenza A H1N1(2009)*. The assay effectiveness was also not affected by endogenous and exogenous interfering substances, such as fresh human blood, mucus, aspirin, and tobramycin. A total number of 262 clinical samples were detected, whose results showed good agreement with RT-PCR and epidemiological investigation results. Since the detection failed in a small number of samples, we further analyzed the results and found that the Ct values of these samples detected by RT-PCR were basically above 35, mainly concentrated around 39, which was below the LOD of ARMS-PCR. More specifically, the LOD of RT-PCR (Sansure Biotech) used in our study was 200 copies/mL, whereas the LOD of the ARMS-PCR method we proposed was 500 copies/mL.

Nowadays, nucleotide sequencing was used to identify SARS-CoV-2 variants. Our developed ARMS assay has several advantages over conventional sequencing. First, it can be completed in less than 90 min and can be synchronized for high-throughput detection. Second, the whole experiment operation is equivalent to RT-PCR, which is simple and easy to implement in a routine laboratory. Third, for the interpretation of results, only computation of the cut-off values is necessary, whereas no complicated analysis comparing sequences is required, which saves manpower and material resources. Last, the ARMS-PCR method can directly use the existing conventional RT-PCR instruments in the laboratory, unlike sequencing, which needs to buy an expensive sequencer or send it to a third party for testing. Therefore, compared with the sequencing method, our method has the advantages of lower cost, faster detection, stronger availability, and more convenience (Simner et al., 2018; John et al., 2021). In fact, due to resource constraints and unequal distribution, sequencing can be used only for the detection of a small number of samples, even in countries with high sequencing capacity. Nevertheless, the method proposed here can be used for the detection of a large number of samples, requiring only a conventional PCR-level laboratory. In addition, our method can be used for molecular-level identification of samples with low viral loads that are difficult to meet the requirements of sequencing. In addition, compared with routine RT-PCR, this assay not only could detect SARS-CoV-2 but performed also preliminarily identification of its variant. A number of detection methods have been developed to identify novel coronavirus mutant types. For example, Garson et al. introduced double-mismatch allele-specific real-time reverse transcription PCR for the detection of SARS-COV-2 Delta variants, but only for the L452R and T478K sites of S spike (Garson et al., 2022). Further, Fabiani et al. reported a rapid and low-cost technique to distinguish the Alpha, Beta, Gamma, and Delta SARS-CoV-2 variants by detecting spike gene mutations using RT-PCR (Fabiani et al., 2022). However, all the samples in their study had a cycle threshold (Ct) value less than or equal to 30, implying uncertainty with Ct values above 30. Notably, 73 samples analyzed in our study had Ct values over 30 and only 10 samples that had Ct values over 35 were not successfully detected.

Nonetheless, our study still has a few shortcomings. Due to sample collection, most of the clinical samples used in this study were swabs, and the comparison with other sample types was lacking. Another concern of this approach is that due to the excessive mutation sites of *S*-gene, the relatively conservative *ORF1ab* gene was used as a substitute for internal control. One important future direction is to design *S*-gene as the internal control to improve the sensitivity and specificity of this method. Another significant future direction is to explore the use of the assay in different types of clinical samples, such as sputum. The experimental results in this study will facilitate the designing of mutant primers and developing ARMS to detect the latest variants, such as Omicron.

In conclusion, the proposed ARMS method can be used for rapid, sensitive, and specific identification of the B.1.617 lineage, especially the Delta variant. This novel method is simple, economical, and can be implemented in a conventional PCR laboratory, saving time and personnel costs.

## Data availability statement

The original contributions presented in the study are included in the article/**Supplementary Material**. Further inquiries can be directed to the corresponding authors.

## Ethics statement

The studies involving human participants were reviewed and approved by the Medical Ethics Committee of the Second Hospital of Nanjing (2021-LS-ky037). Written informed consent for participation was not required for this study in accordance with the national legislation and the institutional requirements. All samples used in this study were from the patient’s leftover swabs after clinical inspection and did not involve the patient’s privacy. Patient information collected in the case system did not contain name, address or other personal information, so the patient’s written informed consent was exempt. The study was conducted in accordance with Declaration of Helsinki.

## Author contributions

Conceptualization: XW, RoZ. Methodology: XW, RoZ. Funding acquisition: XW. Project administration: XW, QZ, RoZ. Writing – original draft: QZ, XW. Writing – review and editing: XW, RoZ. Sample collection: MS, GT, KS, LX. Nucleic acid extraction: QZ, GT, KS, NW. Experimental operation: QZ, RuZ, YG, ZL. Material preparation: QZ, SL, LP, SZ. Manuscript Revision: XW, QZ, RQ, JN, XX. Data collection and analysis: RQ, JN, XX. All authors contributed to the article and approved the submitted version.
