# Supplementary material for: Evaluation of an identification method for the SARS-CoV-2 Delta variant based on the amplification-refractory mutation system
Source: Front Cell Infect Microbiol. 2023 Jul 5;13:1180297. doi: 10.3389/fcimb.2023.1180297 (PMC10354518; doi:10.3389/fcimb.2023.1180297)
Supplement: Supplementary file 1 [file Table_1.docx]

**Supplementary Tables:**

**Table S1.** The sequences of the positive and negative controls.

| Positive control | RNaseP | 5′ CATGGAGAAACTAGAAAAACTGCTTTTGGAATTATCTCTACAGTG  AAGAAACCTCGGCCATCAGAAGGAGATGAAGATTGTCTTCCAGCTT  CCAAGAAAGCCAAGTGGTCTTGCTTTGTTGCTCAGGCTGGAGTACA  GTGGCATAATCTCGGCTCACTGCAACCTTTGCCTCTTGGGCTCAAGC  CATCCTCCCACCTCAGCCTCCCAAGAACTAGAATTCAACAAAGACA  ACTTTTGATCTCTCATCAGAGAGATCATACTCCCAAGAACAGGCTTT  GACCCTTCTTTAAAAGGTTGGTACCCAAGTATACATTTATCTGTTTTA  AGCTCAAAATGCTTAACAGGATGT |
| --- | --- | --- |
|  | The target plasmid | 5′ ACTTACTCCTACTTGGCGTGTTTATTCTACAGGTTCTAATGTTTTTC  AAACACGTGCAGGCTGTTTAATAGGGGCTGAACATGTCAACAACTC  ATATGAGTGTGACATACCCATTGGTGCAGGTATATGCGCTAGTTATCA  GACTCAGACTAATTCTCATCGGCGGGCACGTAGTGTAGCTAGTCAAT  CCATCATTGCCTACACTATGTCACTTGGTGCAGAAAATTCAGTTGCT  TACTCTAATAACTCTATTGCCATACCCACAAATTTTACTATTAGTGTTA  CCACAGAAATTCTACCAGTGTCTATGACCCATTTGTAATTAGAGGTG  ATGAAGTCAGACAAATCGCTCCAGGGCAAACTGGAAAGATTGCTG  ATTATAATTATAAATTACCAGATGATTTTACAGGCTGCGTTATAGCTT  GGAATTCTAACAATCTTGATTCTAAGGTTGGTGGTAATTATAATTACC  GGTATAGATTGTTTAGGAAGTCTAATCTCAAACCTTTTGAGAGAGAT  ATTTCAACTGAAATCTATCAGGCCGGTAGCACACCTTGTAATGGTGT  TCAAGGTTTTAATTGTTACTTTCCTTTACAATCATATGGTTTCCAACC  CACTAATGGTGTTGGTTACCAACCATACAGAGTAGTAGTACTTTCTT  TTGAACTTCTACATGCACCAGCAACTGTTTGTGGACCTAAAAAGTC  TACTAATTTGGTTAAAAACAAATGTGTCAATTTCAACTTCAATGTAC  CACTTCAGAGAGCTAGGTGTTGTACATAATCAGGATGTAAACTTACA  TAGCTCTAGACTTAGTTTTAAGGAATTACTTGTGTATGCTGCTGACCC  TGCTATGCACGCTGCTTCTGGTAATCTATTACTAGATAAACGCACTAC  GTGCTTTTCAGTAGCTGCACTTACTAACAATGTTGCTTTTCAAACTG  TCAAACCCGGTAATTTTAACAAAGACTTCTATGACTTTGCTGTGTCT  AAGGGTTTCTTTAAGGAAGGAAGTTCTGTTGAATTAAAACACTTCT  TCTTTGCTCAGGATGGTAATGCTGCTATCAGCGATTATGACTACTATC  GTTATAATCTACCAACAATGTGTGATATCAGACAACTACTATTTGTAG  TTGAAGTTGTTGATAAGTACTTTGATTGTTACGATGGTGGCTGTATTA  ATGCTAACCAAGTCATCGTC |
| Negative control | RNasep | 5′CATGGAGAAACTAGAAAAACTGCTTTTGGAATTATCTCTACAGTG  AAGAAACCTCGGCCATCAGAAGGAGATGAAGATTGTCTTCCAGCT  TCCAAGAAAGCCAAGTGGTCTTGCTTTGTTGCTCAGGCTGGAGTA  CAGTGGCATAATCTCGGCTCACTGCAACCTTTGCCTCTTGGGCTCA  AGCCATCCTCCCACCTCAGCCTCCCAAGAACTAGAATTCAACAAA  GACAACTTTTGATCTCTCATCAGAGAGATCATACTCCCAAGAACAG  GCTTTGACCCTTCTTTAAAAGGTTGGTACCCAAGTATACATTTATCT  GTTTTAAGCTCAAAATGCTTAACAGGATGT |

**Table S2.** Primer concentration and dosage of each detection site.

| **Detection site** | **Primer** | **Concentration** | **Dosage/per** |
| --- | --- | --- | --- |
| L452R | L452R-F | 400nmol/L | 0.1μL |
|  | L452R-R | 400nmol/L | 0.1μL |
|  | L452R-P-FAM | 200nmol/L | 0.05μL |
| P681R | P681R-F | 400nmol/L | 0.1μL |
|  | P681R-R | 400nmol/L | 0.1μL |
|  | P681R-P-VIC | 200nmol/L | 0.05μL |
| RNaseP | RNaseP-F | 400nmol/L | 0.1μL |
|  | RNaseP-R | 400nmol/L | 0.1μL |
|  | RNaseP-P-CY5 | 200nmol/L | 0.05μL |
| E484Q | E484Q-F | 600nmol/L | 0.15μL |
|  | E484Q-R | 600nmol/L | 0.15μL |
|  | E484Q-P-VIC | 300nmol/L | 0.075μL |
| ORF1ab | ORF1ab-F | 400nmol/L | 0.1μL |
|  | ORF1ab-R | 400nmol/L | 0.1μL |
|  | ORF1ab-P-FAM | 200nmol/L | 0.05μL |

**Table S3.** Information of cross-pathogens used in cross-reactivity.

| **Pathogens** | **Material** | **Concentration** |
| --- | --- | --- |
| *Coronavirus 229E* | RNA | 50ng/μL |
| *Coronavirus OC43* | RNA | 50ng/μL |
| *Coronavirus HKU1* | RNA | 50ng/μL |
| *Coronavirus NL63* | RNA | 50ng/μL |
| SARS-CoV-2 | RNA | 1.5×10^3^copies/mL |
| SARS-coronavirus | VLP | 1×10^6^copies/mL |
| MERS-coronavirus | VLP | 1×10^6^copies/mL |
| *Adenovirus 1* | Culture | 1×10^5^ PFU/mL |
| *Adenovirus 2* | Culture | 1×10^5^ PFU/mL |
| *Adenovirus 3* | Culture | 1×10^5^ PFU/mL |
| *Adenovirus 4* | Culture | 1×10^5^ PFU/mL |
| *Adenovirus 5* | Culture | 1×10^5^ PFU/mL |
| *Adenovirus 7* | Culture | 1×10^5^ PFU/mL |
| *Adenovirus 14* | Culture | 1×10^5^ PFU/mL |
| *Adenovirus 55* | Culture | 1×10^5^ PFU/mL |
| *Adenovirus 71* | Culture | 1×10^5^ PFU/mL |
| *Human Metapneumovirus (hMPV)* | Culture | 1×10^5^ PFU/mL |
| *Parainfluenza virus 1* | Culture | 1×10^5^ PFU/mL |
| *Parainfluenza virus 2* | Culture | 1×10^5^ PFU/mL |
| *Parainfluenza virus 3* | Culture | 1×10^5^ PFU/mL |
| *Influenza A H1N1(2009)* | Culture | 1×10^5^ PFU/mL |
| *Influenza A H3N2* | Culture | 1×10^5^ PFU/mL |
| *Influenza B Yamagata &Victoria* | Culture | 1×10^5^ PFU/mL |
| *Enterovirus 71* | Culture | 1×10^5^ PFU/mL |
| *Respiratory syncytial virus A* | Culture | 1×10^5^ PFU/mL |
| *Respiratory syncytial virus B* | Culture | 1×10^5^ PFU/mL |
| *Rhinovirus A* | Culture | 1×10^5^ PFU/mL |
| *Rhinovirus B* | Culture | 1×10^5^ PFU/mL |
| *Rhinovirus C* | Culture | 1×10^5^ PFU/mL |
| *Chlamydia pneumoniae* | Culture | 1×l0^6^ CFU/mL |
| *Haemophilus influenza* | Culture | 1×l0^6^ CFU/mL |
| *Legionella pneumophila* | Culture | 1×l0^6^ CFU/mL |
| *Mycoplasma pneumoniae* | Culture | 1×l0^6^ CFU/mL |
| *Streptococcus pneumoniae* | Culture | 1×l0^6^ CFU/mL |
| *Streptococcus pyogenes* | Culture | 1×l0^6^ CFU/mL |
| *Bordetella pertussis* | Culture | 1×l0^6^ CFU/mL |
| *Mycobacterium tuberculosis* | Culture | 1×l0^6^ CFU/mL |

**Table S4.** The potential endogenous and exogenous interfering substances.

| **Endogenous interfering substances** | **Concentration** | **Exogenous interfering substances** | **Concentration** |
| --- | --- | --- | --- |
| Fresh human blood | 5% | α- interferon | 100 units/mL |
| Nasal secretions | 5% | Zanamivir | 5mg/L |
| Mucus | 5% | Ribavirin | 0.2g/L |
| Mucoprotein | 2g/dL | Mupirocin | 0.2% |
| / | / | Aspirin | 50mg/L |
| / | / | Tobramycin | 10mg/L |

**Table S5.** Youden’s index of cut-off values of L452R, E484Q, and P681R mutations on the *S*-gene and *ORF1ab* gene of SARS-CoV-2.

| **Object** | **Critical** | **Sensitivity** | **Specificity** | **Youden index** |
| --- | --- | --- | --- | --- |
| L452R | 40.0 | 99% | 100% | 99% |
| E484Q | 40.0 | 99% | 100% | 99% |
| P681R | 40.0 | 96% | 100% | 96% |
| *ORF1ab* | 40.0 | 100% | 100% | 100% |

**Table S6.** Youden’s index of cut-off ∆Ct values of L452R, E484Q, and P681R mutations on the *S*-gene of SARS-CoV-2.

| **Object** | **Critical** | **Sensitivity** | **Specificity** | **Youden index** |
| --- | --- | --- | --- | --- |
| L452R | 10.0 | 100% | 94% | 94% |
| E484Q | 4.0 | 100% | 96% | 96% |
| P681R | 10.0 | 100% | 94% | 94% |

**Table S7.** Determination of LOD by nasopharyngeal swabs, throat swabs, and sputum.

| **Sample ID** | **Positive results (n)/Negative results (n)** | | | | |
| --- | --- | --- | --- | --- | --- |
|  | **5 × 10^5^** | **5 × 10^4^** | **5 × 10^3^** | **5 × 10^2^** | **5 × 10^1^** |
| Sample 1 | 20/0 | 20/0 | 20/0 | 20/0 | 3/17 |
| Sample 2 | 20/0 | 20/0 | 20/0 | 20/0 | 1/19 |
| Sample 3 | 20/0 | 20/0 | 20/0 | 20/0 | 1/19 |
| Sample 4 | 20/0 | 20/0 | 20/0 | 19/1 | 0/20 |
| Sample 5 | 20/0 | 20/0 | 20/0 | 20/0 | 2/18 |
| Sample 6 | 20/0 | 20/0 | 20/0 | 20/0 | 1/19 |

**Table S8.** Results of cross-reactivity with cross-pathogens.

| **Pathogens** | **Concentration** | **Result (No. Positive /**  **No. Tested)** | | **Result** |
| --- | --- | --- | --- | --- |
|  |  | **Negative samples** | **3×LOD** |  |
| *Coronavirus 229E* | 50ng/μL | 0/3 | 3/3 | Non-reactive |
| *Coronavirus OC43* | 50ng/μL | 0/3 | 3/3 | Non-reactive |
| *Coronavirus HKU1* | 50ng/μL | 0/3 | 3/3 | Non-reactive |
| *Coronavirus NL63* | 50ng/μL | 0/3 | 3/3 | Non-reactive |
| SARS-CoV-2 | 1.5×10^3^ copies/mL | 0/3 | 3/3 | Non-reactive |
| SARS | 1×10^6^ copies/mL | 0/3 | 3/3 | Non-reactive |
| MERS | 1×10^6^ copies/mL | 0/3 | 3/3 | Non-reactive |
| *Adenovirus 1* | 1×10^5^ PFU/mL | 0/3 | 3/3 | Non-reactive |
| *Adenovirus 2* | 1×10^5^ PFU/mL | 0/3 | 3/3 | Non-reactive |
| *Adenovirus 3* | 1×10^5^ PFU/mL | 0/3 | 3/3 | Non-reactive |
| *Adenovirus 4* | 1×10^5^ PFU/mL | 0/3 | 3/3 | Non-reactive |
| *Adenovirus 5* | 1×10^5^ PFU/mL | 0/3 | 3/3 | Non-reactive |
| *Adenovirus 7* | 1×10^5^ PFU/mL | 0/3 | 3/3 | Non-reactive |
| *Adenovirus 14* | 1×10^5^ PFU/mL | 0/3 | 3/3 | Non-reactive |
| *Adenovirus 55* | 1×10^5^ PFU/mL | 0/3 | 3/3 | Non-reactive |
| *Adenovirus 71* | 1×10^5^ PFU/mL | 0/3 | 3/3 | Non-reactive |
| *Human Metapneumovirus (hMPV)* | 1×10^5^ PFU/mL | 0/3 | 3/3 | Non-reactive |
| *Parainfluenza virus 1* | 1×10^5^ PFU/mL | 0/3 | 3/3 | Non-reactive |
| *Parainfluenza virus 2* | 1×10^5^ PFU/mL | 0/3 | 3/3 | Non-reactive |
| *Parainfluenza virus 3* | 1×10^5^ PFU/mL | 0/3 | 3/3 | Non-reactive |
| *Influenza A H1N1（2009）* | 1×10^5^ PFU/mL | 0/3 | 3/3 | Non-reactive |
| *Influenza A H3N2* | 1×10^5^ PFU/mL | 0/3 | 3/3 | Non-reactive |
| *Influenza B Yamagata &Victoria* | 1×10^5^ PFU/mL | 0/3 | 3/3 | Non-reactive |
| *Enterovirus 71* | 1×10^5^ PFU/mL | 0/3 | 3/3 | Non-reactive |
| *Respiratory syncytial virus A* | 1×10^5^ PFU/mL | 0/3 | 3/3 | Non-reactive |
| *Respiratory syncytial virus B* | 1×10^5^ PFU/mL | 0/3 | 3/3 | Non-reactive |
| *Rhinovirus A* | 1×10^5^ PFU/mL | 0/3 | 3/3 | Non-reactive |
| *Rhinovirus B* | 1×10^5^ PFU/mL | 0/3 | 3/3 | Non-reactive |
| *Rhinovirus C* | 1×10^5^ PFU/mL | 0/3 | 3/3 | Non-reactive |
| *Chlamydia pneumoniae* | 1×l0^6^ CFU/mL | 0/3 | 3/3 | Non-reactive |
| *Haemophilus influenza* | 1×l0^6^ CFU/mL | 0/3 | 3/3 | Non-reactive |
| *Legionella pneumophila* | 1×l0^6^ CFU/mL | 0/3 | 3/3 | Non-reactive |
| *Mycobacterium tuberculosis* | 1×l0^6^ CFU/mL | 0/3 | 3/3 | Non-reactive |
| *Streptococcus pneumoniae* | 1×l0^6^ CFU/mL | 0/3 | 3/3 | Non-reactive |
| *Streptococcus pyogenes* | 1×l0^6^ CFU/mL | 0/3 | 3/3 | Non-reactive |
| *Bordetella pertussis* | 1×l0^6^ CFU/mL | 0/3 | 3/3 | Non-reactive |
| *Mycoplasma pneumoniae* | 1×l0^6^ CFU/mL | 0/3 | 3/3 | Non-reactive |

**Table S9.** Details of 10 samples with inconsistent results from ARMS-PCR and the RT-PCR test results.

| **Original sample ID** |  | **Ct value of RT-PCR** | | **ARMS-PCR result** |
| --- | --- | --- | --- | --- |
|  |  | *ORF1ab* | [Internal](javascript:;) [reference](javascript:;) |  |
| 20210729COV046 |  | 36 | 26 | Negative |
| 20210729COV057 |  | 38 | 25 | Negative |
| 20210729COV064 |  | 40 | 25 | Negative |
| 20210730COV008 |  | 37 | 27 | Negative |
| 20210730COV009 |  | 36 | 23 | Negative |
| 20210801COV042 |  | 40 | 24 | Negative |
| 20210801COV043 |  | 39 | 24 | Negative |
| 20210804COV113 |  | 39 | 24 | Negative |
| 20210804COV117 |  | 39 | 25 | Negative |
| 20210804COV138 |  | 39 | 29 | Negative |
